# Micro-accelerometer Based on Vertically Movable Gate Field Effect Transistor

**DOI:** 10.1007/s40820-015-0041-9

**Published:** 2015-05-06

**Authors:** Heung Seok Kang, Kang-Hee Lee, Dong-Youk Yang, Byoung Hee You, In-Hyouk Song

**Affiliations:** 1 Korea Atomic Energy Research Institute, 150 Deokjin-dong, Yuseong-gu, Daejeon, Republic of Korea; 2 LOGTECH Co. Ltd., 914 Gumgang Hightech Valley 1st, Sungnam-si, Gyeonggi-do Republic of Korea; 3 Department of Engineering Technology, Texas State University, 601 University Dr., San Marcos, TX 78666 USA

**Keywords:** MEMS, Micro-accelerometer, Suspended gate FET, Vertically movable gate FET, VMGFET

## Abstract

A vertically movable gate field effect transistor (VMGFET) is proposed and demonstrated for a micro-accelerometer application. The VMGFET using air gap as an insulator layer allows the gate to move on the substrate vertically by external forces. Finite element analysis is used to simulate mechanical behaviors of the designed structure. For the simulation, the ground acceleration spectrum of the 1952 Kern County Earthquake is employed to investigate the structural integrity of the sensor in vibration. Based on the simulation, a prototype VMGFET accelerometer is fabricated from silicon on insulator wafer. According to current–voltage characteristics of the prototype VMGFET, the threshold voltage is measured to be 2.32 V, which determines the effective charge density and the mutual transconductance of 1.545×10^−8^ C cm^−2^ and 6.59 mA V^−1^, respectively. The device sensitivity is 9.36–9.42 mV g^−1^ in the low frequency, and the first natural frequency is found to be 1230 Hz. The profile smoothness of the sensed signal is in 3 dB range up to 1 kHz.

## Introduction

The advancement of technology to make micro-mechanical structure using semiconductor fabrication processes leads the application of electrical components including transistors, capacitors, and inductors for microelectromechanical systems (MEMS) [1–6]. In terms of integration with electronics, silicon-based MEMS have gained much attention due to the potential applications in wireless communication system, mobile device, structural health monitoring system, etc. [6–8]. One of the active electronic devices for sensing elements is movable gate field effect transistors (FET), whose gate is released from the substrate and free to move [9–12]. The MOSFET-type device contains dielectric layer to electrically insulate between gate electrode and substrate embedding source and drain of FET. The movable gate FET uses air gap as an insulator or a dielectric layer of the FET. The use of air layer allows the gate to move laterally along the channel of FET or vertically to the substrate by external forces such as acceleration or pressure. The former is called the laterally movable gate FET (LMGFET), and the latter is called the vertically movable gate FET (VMGFET) [12, 13]. The oscillation frequency of the sensing element is simply converted to electrical output without additional circuitry. In this paper, a micro-accelerometer using VMGFET is introduced, applicable for low-frequency vibration measurement (<500 Hz).

The state-of-the art technologies for low-frequency vibration sensing applications have been mostly used for capacitive or piezoresistive sensing scheme [14–16] while the research of VMGFET has been more focused on pressure/strain sensors or gas sensors [2, 10]. In order to enhance the sensitivity to vertical motion using VMGFET, Aoyagi proposed nickel proof mass on flexible beam, which required alignment and electroplating to form proof mass [17]. The VMGFET in this paper is demonstrated and characterized using silicon on insulator (SOI) wafer for low-frequency vibration measurement. The resonant structure including moving gate of VMGFET is designed with meander-type beams to improve flexibility in vertical direction, and the long-channel device is used to increase the device sensitivity.

VMGFET is fabricated using standard IC fabrication processes such as UV lithography and silicon dry etching process. Silicon-based suspended microstructures with high quality factors can be applied for frequency-sensitive systems such as accelerometer and gyroscope integrated with electronic systems [18, 19]. The silicon-based process benefits mass production through batch fabrication, and compactness due to small size and IC compatibility.

**Fig. 1:**
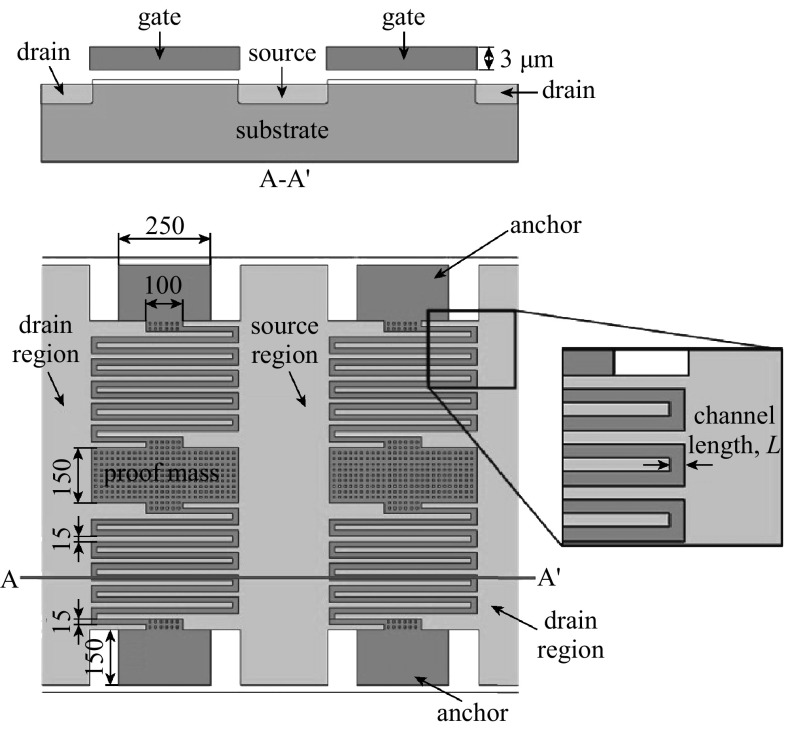
Schematic of the vertically movable gate FET accelerometer (unit: μm). Figure is not drawn to scale

The structural device layer serves as suspension gate and proof mass to induce the movement due to acceleration. Figure 1 is the schematic of the VMGFET accelerometer with a cross-sectional view, which illustrates a serpentine gate structure design with fixed anchors at the ends. The VMGFET structure is obtained by forming an air gap and using SiO_2_ as an insulator layer. The air gap allows the suspended gate to move vertically. The displacement of the gate modulates the charge distribution in the semiconductor and the change in capacitance between gate and substrate [13]. In contrast to the previous VMGFETs or suspended gate FETs, this paper introduces a simple fabrication of VMGFET, which can be applied for low-frequency vibration measurement. This paper describes the electrical properties of fabricated VMGFET and integration of VMGFET with electronics on printable circuit board (PCB), which can be transferred to the monolithic integration in the future.

## Fabrication

**Fig. 2:**
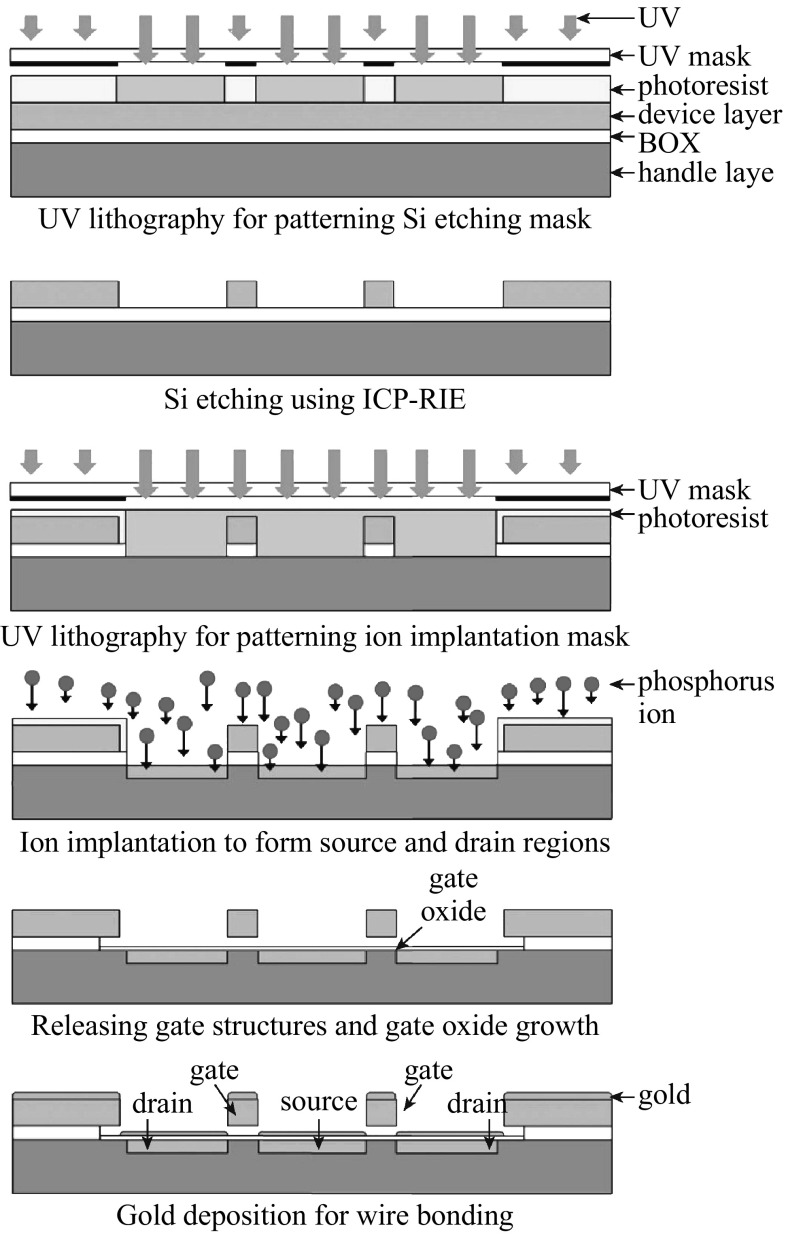
Schematic of fabrication process for the VMGFET accelerometer

**Fig. 3:**
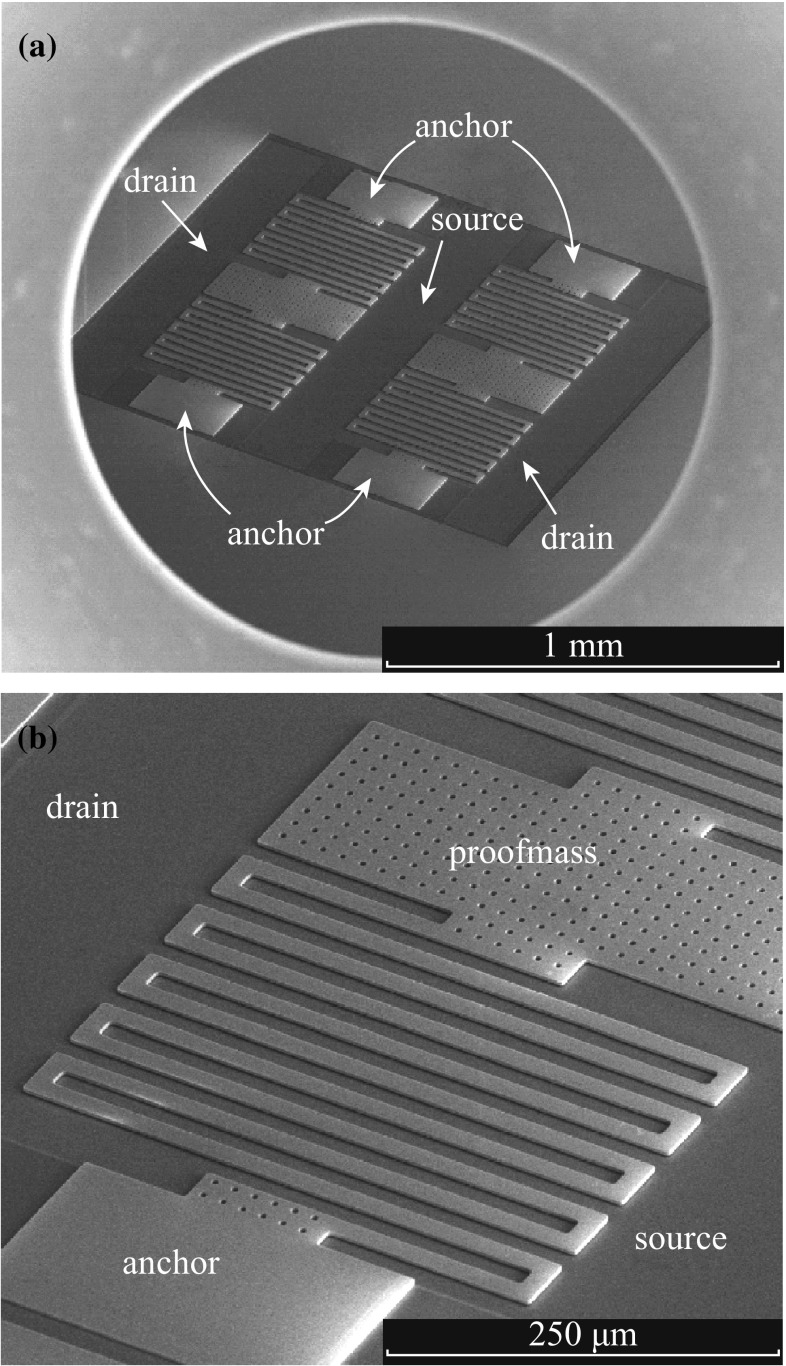
**a** SEM image of fabricated dual VMGFETs. **b** The close-up view of suspended serpentine gate

The process details have been previously described [20]. The basic process to fabricate the VMGFETs consists of two UV lithography steps for silicon dry etching mask and an ion implantation mask shown schematically in Fig. 2. The VMGFETs are prepared using (100) oriented SOI wafers with a 3-µm-thick n-type device layer, a 1-µm-thick buried oxide (BOX) layer, and a 500-µm-thick p-type handle layer. The p-type substrate is selected to form an n-channel MOSFET device. A 3-μm-thick top device layer of SOI wafer is used to fabricate gate structures. The SOI device layer is patterned with the first UV mask using standard UV lithography. The wafer is etched through the device layer to the BOX using ICP-RIE process. A 1-μm-thick BOX layer is then opened and UV patterned using the second UV mask for ion implantation mask patterns. Then, phosphorus ions are implanted in the opened gate structures and the opened p-type substrate to form source and drain regions. Vapor HF etches the sacrificial layer underneath gate structures followed by thermal oxidation on the entire wafer to grow a 33-nm-thick gate oxide of the FET. A 200-nm-thick gold film is deposited on top of the wafer for wire bonding. Figure 3a shows an SEM image of fabricated dual VMGFETs with a common source region. The close-up view of suspended gate details is shown in Fig. 3b.

## Mechanical Analysis

**Fig. 4:**
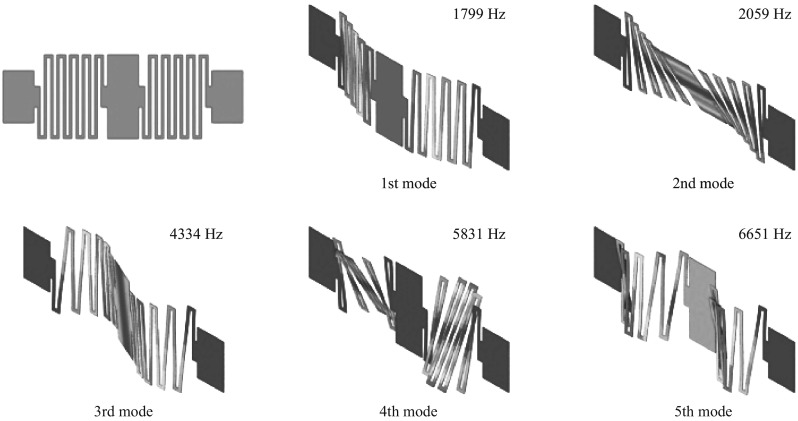
Finite element model and analysis results of the serpentine gate for the first five resonant frequency modes assuming a composite material as silicon–gold

A commercial finite element code, ABAQUS CAE 6.10-1, is used to build a finite element (FE) model and to investigate vibration behavior of the serpentine gate for design verification. Figure 4 shows the FE analysis results obtained assuming that the gate is a homogeneous medium made by silicon–gold composite and the anchors at the end of the gate are defined as fixed constraints. The gate structure is discretized into a finite number of elements in triangular and rectangular shape. Material properties of analysis model referred for the coated gold and silicon are summarized in Table 1. Coating layer of gold is modeled as uniformly distributed mass over the surface of the moving gate, ignoring additional stiffness from bonding interface between the two materials. The FE analysis performs eigenvalue extraction to calculate the natural frequencies and the corresponding mode shapes of the structure. As shown in Fig. 4, the fundamental mode shape of the gate structure is a typical bending mode of plates. The first natural frequency at the mode is obtained at 1799 Hz. The second mode is a twist mode at 2059 Hz, the third mode is the second bending at 4334 Hz, and the fourth mode is the second twist at 5831 Hz, and the fifth mode is the third bending mode at 6651 Hz. The first natural frequency, therefore, is much higher than the applicable range of MEMS sensor of 500 Hz, so that the natural frequencies of the structures of the sensor itself can hardly be affected by the measuring signal.

**Table 1:** Material properties for gold and silicon for numerical model

Material	Property	
Gold	Density	19.2 g cm^−3^
	Measured thickness	0.292 µm
Silicon	Density	2.329 g cm^−3^
	Elastic modulus	169 GPa
	Poison ratio	0.048

Twofold of gravity (2 g) is set as the target base acceleration for the developing sensor. The ground acceleration spectrum of the 1952 Kern County Earthquake, which is commonly used as a reference signal for nuclear power plant design, and is strongest since the 1906 San Francisco earthquake, is selected to investigate the structural integrity of the sensor in vibration [21]. As observed commonly in seismic accidents, most of the energy narrowly spread out within 50 Hz.

**Fig. 5:**
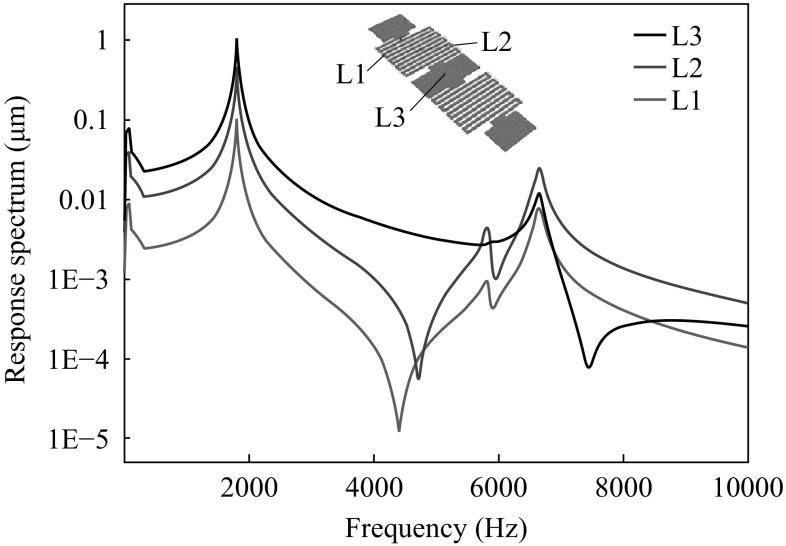
Displacement spectrum corresponding to the ground acceleration spectrum at 1952 Kern County Earthquake in California USA

**Fig. 6:**
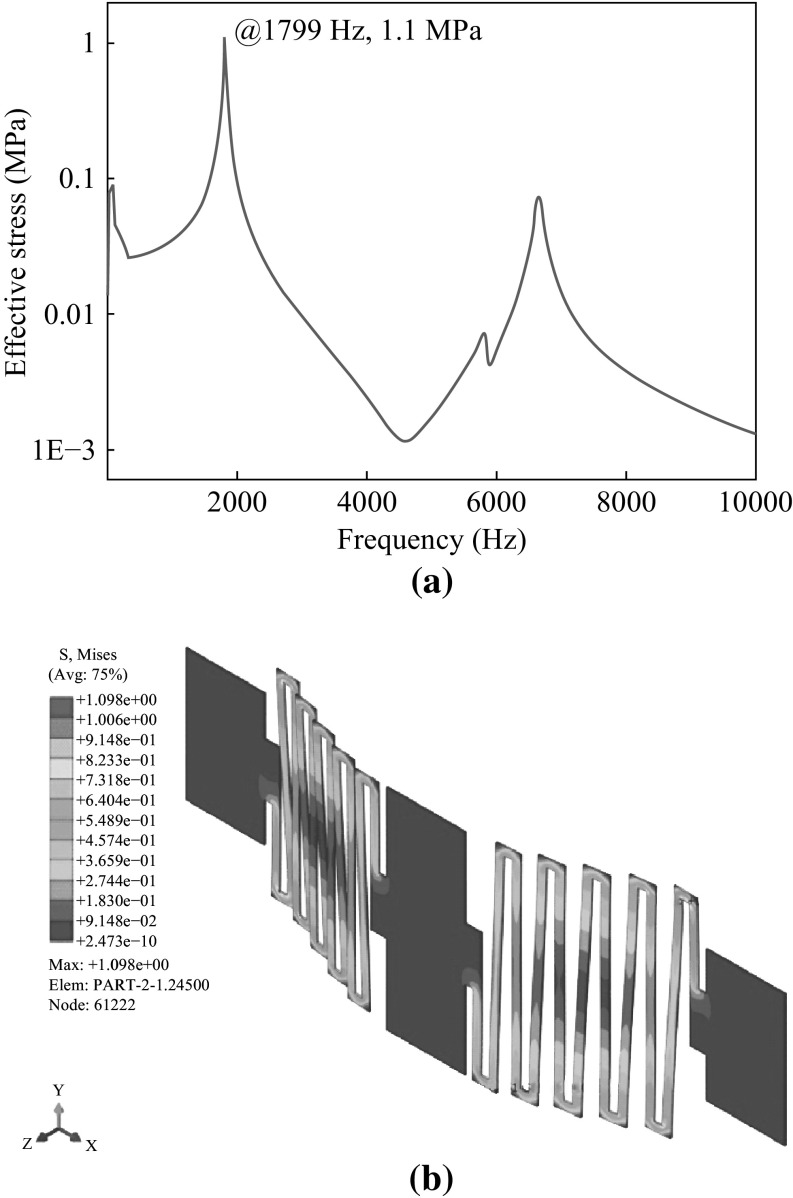
**a** Stress spectrum corresponding to the displacement spectrum of the sensor. **b** Stress field on the serpentine gate calculated by FE analysis while the sensor suffers the 1952 Kern County Earthquake in California USA

Figure 5 shows the displacement response spectrum of the sensing element corresponding to the 1952 Kern Country earthquake spectrum. The first two peaks are the seismic source corresponding spectrum and the first bending mode at the first natural frequency at 10 Hz and 1799 kHz, respectively. The movements of sensing element from the neutral position corresponding to the two peaks in the response spectrum can reach to 0.1 and 1 μm. Figure 6a demonstrates the stress values under the 1952 Kern County Earthquake. Figure 6b shows the stress level and stress field of the structure at 1799 Hz during vibration. The highest stress of 1.1 MPa, which is way below the material strength limit of sensing element, occurs at the first inner edge of the serpentine beam from both the anchors.

## Electrical Analysis

The gate electrode of VMGFET is formed with a 0.1 Ω cm resistivity n-type device layer of SOI wafer. A (100) oriented boron-doped handle layer of SOI wafer exhibits p-type conductivity with 1–20 Ω cm used for a substrate of FET embedding source and drain regions. A 1-μm-thick air gap separates the gate and the substrate. The air gap allows the gate structure of VMGFET to move in vertical direction when the external forces such as acceleration or pressure are applied to the system. The gate motion shifts the current of the FET resulting from the change in air gap. Hence, VMGFET converts acceleration to electrical forms such as current or voltage without any additional circuitry.

For an n-channel VMGFET, a simplified model based on long-channel FET theory gives the drain current, *I*_DS_, in non-saturation region as

1 $$I_{\text{DS}} = \frac{{\mu_{n} W}}{L}C_{\text{eff}}^{'} \left[ {\left( {V_{\text{GS}} - V_{\text{Th}} } \right)V_{\text{DS}} - \frac{1}{2}V_{\text{DS}}^{2} } \right],$$

and in saturation region as

2 $$I_{\text{DS}} = \frac{{\mu_{n} W}}{2L}C_{\text{eff}}^{'} \left( {V_{\text{GS}} - V_{\text{Th}} } \right)^{2} ,$$

where *L* is the channel length, *W* is the gate width over the channel, and *μ*_*n*_ is channel carrier mobility. *V*_GS_ and *V*_DS_ are the potential differences of gate and drain to source, respectively. *V*_Th_ is the threshold voltage to form a channel between source and drain. $C_{\text{eff}}^{'}$ indicates an effective capacitance per unit area between gate electrode and silicon substrate consisting of two dielectrics: $C_{\text{ox}}^{'}$ for gate oxide and $C_{\text{air}}^{'}$ for air. Hence, $C_{\text{eff}}^{'}$ is given by

3 $$C_{\text{eff}}^{'} = \frac{{C_{\text{ox}}^{'} C_{\text{air}}^{'} }}{{C_{\text{ox}}^{'} + C_{\text{air}}^{'} }} = \frac{{\varepsilon_{0} \varepsilon_{\text{rox}} }}{{z_{\text{gap}}\varepsilon_{\text{rox}} + t_{\text{ox}} }},$$

where *ε*_rox_ = 3.9 is the relative dielectric permittivity of gate oxide, *ε*_0_ = 8.854 × 10^−14^ F cm^−1^ is the permittivity of free space, *z*_gap_ is the air gap between the bottom of the moving gate and top of the gate oxide layer over the channel, and *t*_ox_ is the thickness of the oxide. Since, as shown in Eq. (3), $C_{\text{eff}}^{'}$ is inversely proportional to the value of *z*_gap_, the gate motion modulates the drain current in both non-saturation region and saturation region.Threshold voltage, *V*_Th_, of (1) and (2) is given by

4 $$V_{\text{Th}} = \phi_{\text{MS}} + 2\phi_{\text{F}} - \frac{{\left( {Q_{\text{eff}}^{'} + Q_{\text{d}}^{'} } \right)}}{{C_{\text{eff}}^{'} }}.$$

Here *ϕ*_MS_ is the metal–semiconductor work function potential difference. The work functions in heavily doped n-type Si gate electrode and p-type Si substrate of 10 Ω cm resistivity are 4.58 and 5.03 eV, respectively [22]. Hence, *ϕ*_MS_ of the fabricated VMGFET is −0.45 V. A negative value for *ϕ*_MS_ means the gate of a MOS structure is positively charged and the silicon surface is negatively charged at equilibrium. For a p-type Si substrate, potential *ϕ*_F_ is given by

5 $$\phi_{\text{F}} = \frac{kT}{q}\ln \frac{{N_{\text{A}} }}{{n_{i} }},$$

where *q* = 1.6×10^−19^ C is magnitude of electron charge, *k* = 1.38×10^−23^ J K^−1^ is the Boltzmann constant, *T* is the temperature, and *n*_*i*_ = 1.5×10^10^ cm^−3^ is the intrinsic doping concentration. The doping concentration, *N*_A_, of boron-doped p-type Si of 10 Ω cm resistivity is 1.5×10^15^ cm^−3^, which determines *ϕ*_F_ of 0.298 V using Eq. (5). The value for (*ϕ*_MS_ + 2*ϕ*_F_) term is 0.146 V. $Q_{\text{d}}^{'}$ is the depletion region charge per unit area in silicon substrate at onset of strong inversion given by $Q_{\text{d}}^{'} = - \sqrt {4\varepsilon_{s} qN_{\text{A}} \phi_{\text{F}} }$ for a p-type substrate. Here, *ε*_s_ = 11.9 is permittivity of silicon. $Q_{\text{d}}^{'}$ for the given *N*_*A*_ of 1.5 × 10^15^/cm^3^ is −1.736×10^−8^ C cm^−2^. $Q_{\text{eff}}^{'}$ in Eq. (4) is an effective charge density in the gate oxide and at the oxide–silicon interface, which is extracted from experimental results in the following section.

## Experimental Results

**Fig. 7:**
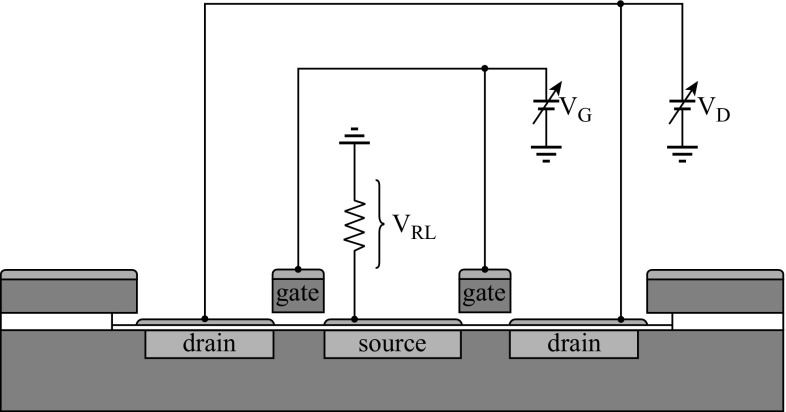
Schematic diagram to measure the DC characteristics of fabricated VMGFET

**Fig. 8:**
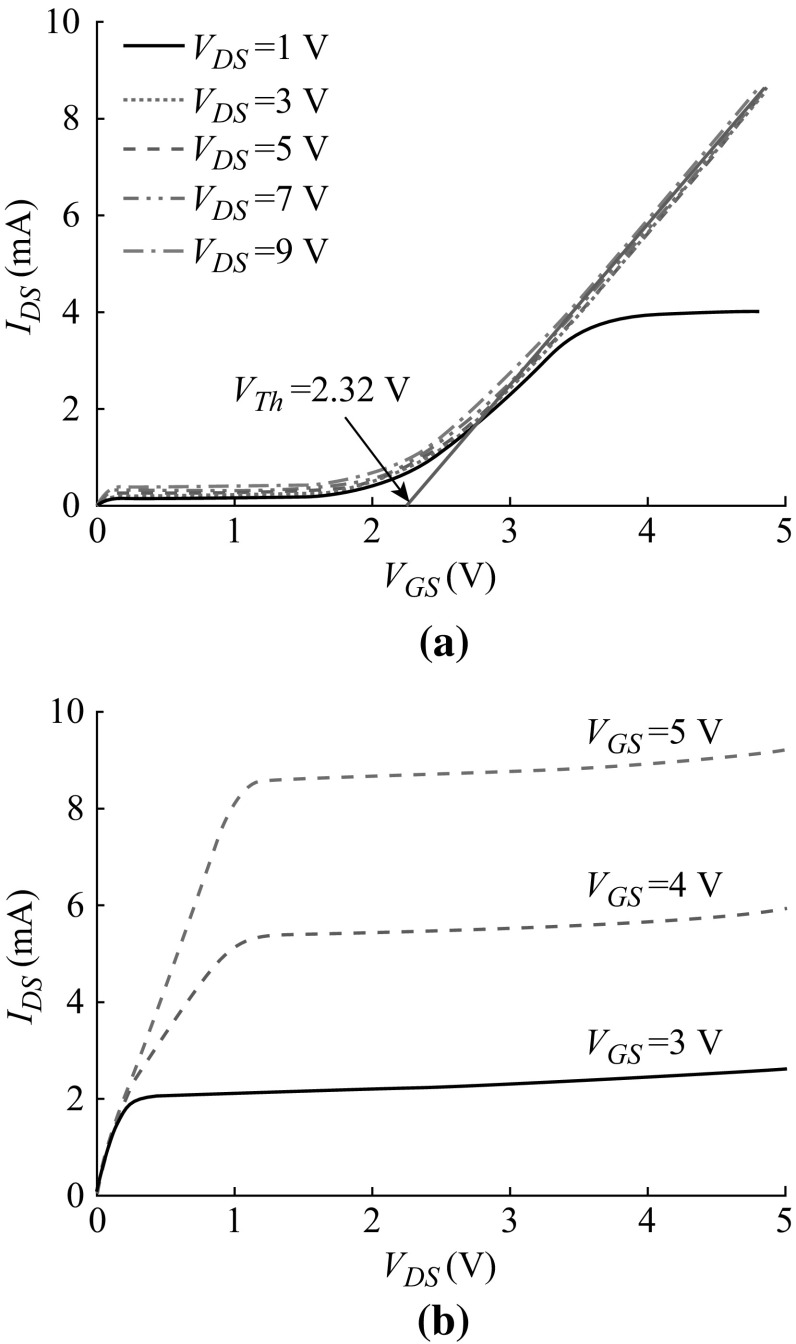
**a** Measured DC *I*_DS_–*V*_GS_ characteristics with five different *V*_DS_. **b** Measured DC *I*_DS_–*V*_DS_ characteristics with three different *V*_GS_

Figure 7 shows the electric measurement design of dual VMGFETs with a common source. The measured DC current–voltage (*I*_DS_–*V*_GS_) characteristics of the prototype of VMGFET are shown in Fig. 8a for five different *V*_DS_ values. The threshold voltage of VMGFET extracted is 2.32 V, which indicates that an enhancement-type n-channel device. $Q_{\text{eff}}^{'}$ of 1.545×10^−8^ C cm^−2^ is extracted from measured *V*_Th_ (= 2.32 V) and $C_{\text{eff}}^{'}$ (= 8.780×10^−10^ F cm^−2^) without gate motion under 1-μm air gap using Eq. (4).

Figure 8b shows the current–voltage (*I*_DS_–*V*_DS_) characteristics of the fabricated n-channel VMGFET device for three different *V*_GS_ values. Using Eq. (2) in saturation region, the mutual transconductance, *g*_msat_, is given as

6 $$g_{\text{msat}} = \left. {\frac{{\partial I_{\text{DS}} }}{{\partial V_{\text{GS}} }}} \right|_{{V_{\text{DS}} \, \ge \,V_{\text{DSsat}} }} \approx \left. {\frac{{\Delta I_{\text{DS}} }}{{\Delta V_{\text{GS}} }}} \right|_{{V_{\text{DS}} \, \ge \,V_{\text{DSsat}} }} = \frac{{\mu_{n} W}}{L}C_{\text{eff}}^{'} \left( {V_{\text{GS}} - V_{\text{Th}} } \right) = \frac{{2I_{\text{DS}} }}{{V_{\text{GS}} - V_{\text{Th}} }}.$$

The measured *V*_Th_ provides the value of *g*_msat_ = 6.59 mA V^−1^ from Fig. 8b which shows *I*_DS_ = 5.535 mA at *V*_DS_ = 3 V and *V*_GS_ = 4 V.

The measured drain current in Fig. 8b is the result under gravity of the earth (+1 g). Then the sensor is inverted 180° and experiences a negative gravity (−1 g). The measured *I*_DS_ under –1 g is decreased to 5.487 mA at *V*_DS_ = 3 V and *V*_GS_ = 4 V, which is 5.535 mA under +1 g. The test shows the current sensitivity of 24 µA g^−1^ at *V*_DS_ = 3 V and *V*_GS_ = 4 V.

**Fig. 9:**
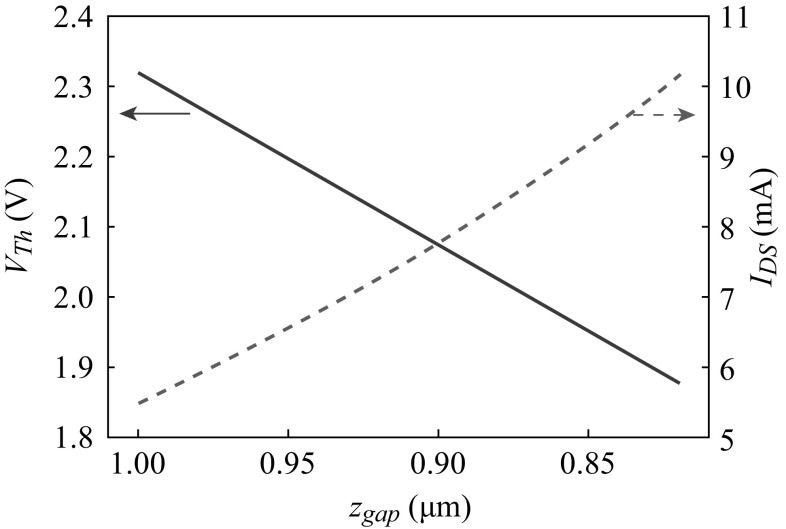
Simulated threshold voltage, *V*_Th_, of the prototype VMGFET based on **(** 3) and (4 **)** and simulated drain current, *I*_DS_, at *V*_*DS*_ = 3 V and *V*_GS_ = 4 V as a function of air gap thickness, *z*_gap_

Figure 9 is a simulation result based on (3) and (4) as a function of air gap distance, *z*_gap_. For this simulation, the following conditions are used: *N*_A_ = 1 × 10^15^/cm^3^, *t*_ox_ = 33 nm, $Q_{\text{d}}^{'}$ = −1.736×10^−8^ C cm^−2^, and $Q_{\text{eff}}^{'}$ = 1.545×10^−8^ C cm^−2^. As shown in Fig. 9, *V*_Th_ is not constant and linearly decreased as the gate is closed to the substrate. With the decreasing air gap, the change in *V*_Th_ of the fabricated VMGFET modulates the drain current in both non-saturation and saturation regions. The value of (*µ*_*n*_ *W* (2*L*)^*−*1^) in Eq. (2) can be extracted from Fig. 8b for the evaluation of device motion sensitivity. The drain current is 5.535 mA at *V*_DS_ = 3 V and *V*_GS_ = 4 V for 1-µm air gap. The threshold voltage and the effective capacitance are 2.32 V and 8.854×10^−10^ F/cm^2^, respectively. Hence, the value of (*µ*_*n*_ *W*/2*L*) is 2.2375×10^6^ cm^2^ (V s)^−1^ from Eq. (2). Figure 9 also shows the simulation of drain current as a function of air gap thickness at *V*_DS_ = 3 V and *V*_GS_ = 4 V. The simulation is based on (2). For example, the 50 nm gate displacement down from the original position increases the drain current to 5.634 mA.

**Fig. 10:**
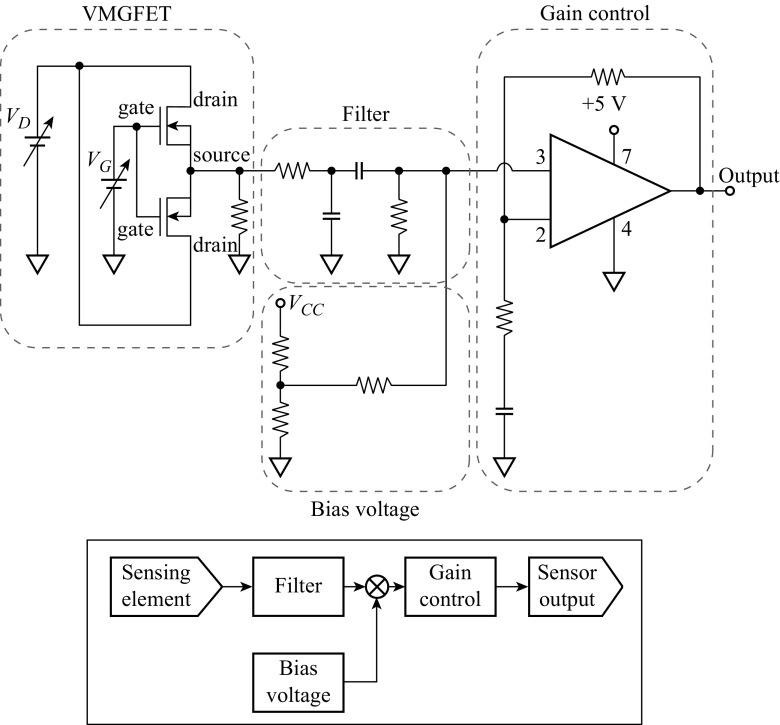
Schematic circuit diagram to measure frequency analysis and device sensitivity

**Fig. 11:**
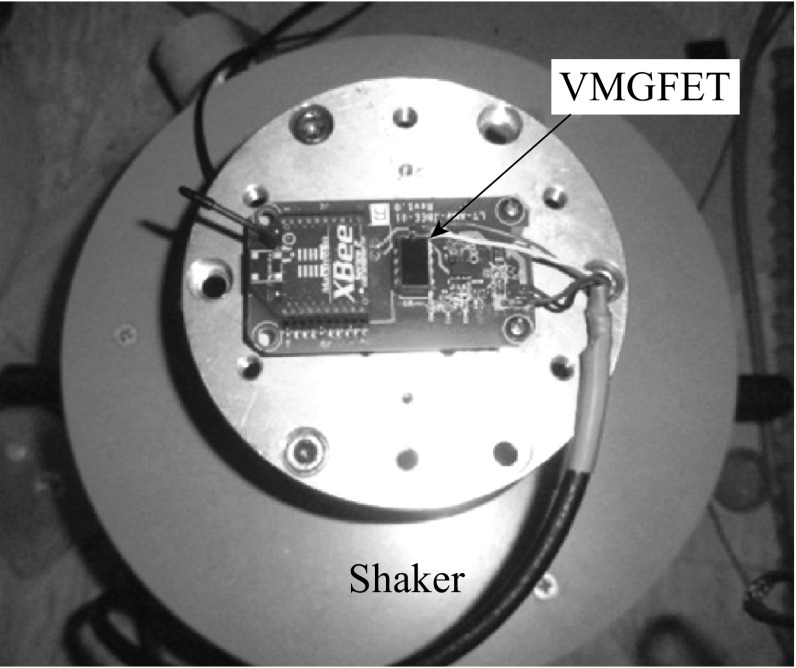
Experimental setup mounted on shaker to apply vibrations to the prototype VMGFET integrated on PCB

The output voltage of VMGFET is measured using readout circuit integrated on a printed circuit board (PCB) to detect voltage changes. Figure 10 illustrates the block diagram of readout circuit and displacement detection circuit with filter and amplifier. The gates move toward or backward the substrate when the acceleration is applied to the common source VMGFETs. The voltage across the load resistor is filtered and amplified. The readout circuit outputs a voltage proportional to the displacement of gate from steady state position. The experiments with external acceleration are performed on a setup comprising a platform with a fixed stage on a vibration exciter as shown in Fig. 11. The shaker actuates the suspended gate vertically since the gate motion is out of plane and the sensitivity axis is aligned with the platform’s vertical direction.

**Fig. 12:**
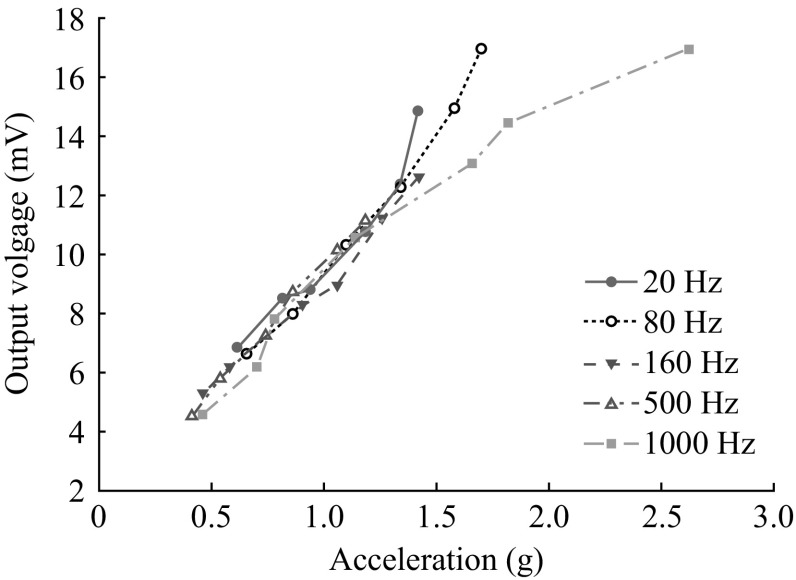
Output response to applied accelerations for different frequencies

**Fig. 13:**
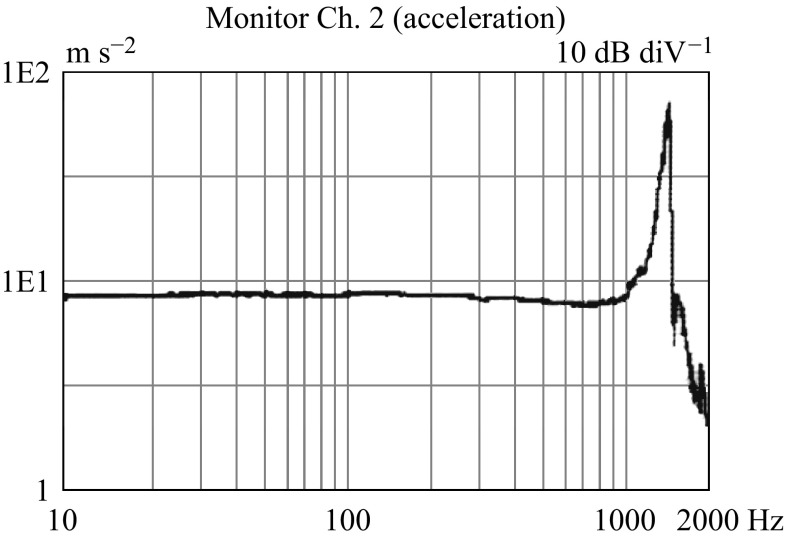
Frequency response of the prototype VMGFET. The resonant frequency is at 1.23 kHz

Figure 12 shows the relation of input acceleration to output voltage for different actuation frequencies. According to the results, the device shows the linear feedback voltage sensitivity of 9.36–9.42 mV g^−1^ in the measurement range of 0.5–1.2 g for the actuated frequencies. The frequency response of vibration measurement provides the flatness of measuring signal along the operating frequency range and the location of the first natural frequency, which determines the operating frequency range of the fabricated VMGFET. The frequency response of the fabricated device and the flatness of the signal are measured using SC-1000 Sine vibration controller (IMV Corp., Japan) as shown in Fig. 13. It shows the first natural frequency of the tested device at 1230 Hz which is a lower resonant frequency than that of simulation. As shown in Fig. 13, the profile smoothness of the sensed signal is in 3 dB range up to 1 kHz. Under a 1 g (= 9.8 m s^−2^) acceleration, the sensed accelerations are 9.78 and 9.15 m s^−2^ at 10 and 500 Hz, respectively.

## Conclusion

A VMGFET is designed and fabricated for a micro-accelerometer, which converts the acceleration to electrical signals. In order to form VMGFET, SOI wafer is used to simplify the fabrication processes. Prior to the fabrication and electrical measurement, a finite element analysis is carried out to evaluate mechanical feasibility and structural integrity as a sensing element. Free vibration analysis shows that the fundamental natural frequency of the gate structure is 1799 Hz whose corresponding mode shape is a typical bending mode of a plate. In this paper, the maximum base excitation for seismic response spectrum analysis is twofold of gravity (2 g). The ground acceleration spectrum of the 1952 Kern County Earthquake, commonly used as the reference excitation signal for nuclear power plant design, is selected to investigate the structural integrity of the sensor in vibration. Highest stress, at the first inner edge of the serpentine beam from both the anchors, from seismic excitation is way below the material strength limit.

By DC electrical measurement, the threshold voltage and the effective charge density in the gate oxide and at the interface between silicon and gate oxide are determined of 2.32 V and 1.545×10^−8^ C cm^−2^, respectively. A readout circuit is designed and integrated on PCB with VMGFET. The voltage sensitivity of the device is 9.36–9.42 mV g^−1^ up to 1000 Hz. According to the frequency spectrum, the first natural frequency of the tested device is located at 1230 Hz, which is lower than that of the simulation result. The difference between the simulation and experiment can be due to the actual thickness of the device layer being thinner than that used for the simulation and the deposited gold film thickness being thicker, which does not provide accurate parameter information for the simulation. The prototype VMGFET shows that the smoothness of the sensed signal profile is in 3 dB range up to 1 kHz. Under a 1 g (= 9.8 m s^−2^) acceleration, the sensed accelerations are 9.78 and 9.15 m s^−2^ at 10 and 500 Hz, respectively. Seismic load is narrowly distributed in the frequency range of 0 to 40 Hz. For the application of seismic monitoring in nuclear power plants, the accelerometer would be sensitive and reliable in the low frequency range. Hence, the prototype VMGFET is suitable for the acceleration measurement of the low-frequency seismic vibrations.
